# Political and Ideological Work in the Southern Battlefield and the Construction of the Vietnamese Revolutionary Image in Foreign Affairs (1954–1975)

**DOI:** 10.12688/f1000research.178080.1

**Published:** 2026-03-13

**Authors:** Nguyen Thi Phuong

**Affiliations:** 1 University of Social Sciences and Humanities, VNU-HCM, Ho Chi Minh City, Vietnam; 2 Vietnam National University, Ho Chi Minh City, Vietnam

**Keywords:** Political and ideological work, Southern Battlefield, Vietnamese revolutionary image, Revolutionary diplomacy, Soft power

## Abstract

**Background:**

The resistance war against the United States was not merely a military struggle but also a diplomatic and international public opinion battle. In this context, constructing the image of the Vietnamese Revolution played a particularly crucial role in consolidating legitimacy and garnering international support. Consequently, the ideological, cultural, and social fronts within revolutionary battlefields became an organic component of the revolutionary foreign policy strategy.

**Research Problem:**

Alongside formal diplomatic activities at the central level, political and ideological work within revolutionary battlefields particularly the Southern battlefield contributed to shaping and disseminating the image of the Vietnamese Revolution to the international public. However, the role of grassroots political-ideological work in image-building and supporting foreign affairs has not yet been systematically examined in existing diplomatic and international relations literature.

**Objectives:**

This article aims to analyze the role of political and ideological work in the Southern battlefield in constructing the Vietnamese revolutionary image for foreign affairs from 1954 to 1975. Through this analysis, the study clarifies the relationship between building domestic political and social strength and consolidating the international image and legitimacy of the Vietnamese Revolution.

**Methods:**

The study employs the historical method combined with document analysis and international relations approaches regarding image, legitimacy, and soft power. Research is based on an examination of party documents, propaganda materials, memoirs, revolutionary journalism, and various international sources related to peace movements and global public opinion on the Vietnam War.

**Main Conclusions:**

The research indicates that political and ideological work in the Southern battlefield did not only serve to build internal revolutionary strength but also acted as a vital resource for constructing the national image, reinforcing legitimacy, and enhancing the diplomatic effectiveness of the Vietnamese Revolution during the anti-American resistance war.

## 1. Introduction

### 1.1 Historical and foreign affairs context

In the study of international relations, the role of soft power which enhances the ability to attract and persuade international partners through culture, values, and policies rather than coercion is considered a vital resource in the diplomatic strategies of states and political movements. According to Joseph Nye, who laid the conceptual foundation for this term, soft power is the ability to achieve goals through attraction and persuasion based on an international actor's cultural and socio-political resources rather than force. Nye popularized this term in his 1990 book, Bound to Lead: The Changing Nature of American Power. In it, he writes: When one country gets other countries to want what it wants might be called co-optive or soft power in contrast to the hard or command power of ordering others to do what it wants ( Nye, 2004). He further developed this concept in his 2004 work, Soft Power: The Means to Success in World Politics.

In the Vietnamese context during the 1954-1975 period, revolutionary diplomacy became a critical strategic front in the resistance war for national unification. The Ministry of Foreign Affairs of Vietnam and various historical studies emphasize that diplomacy during this era was not merely discourse between states; it was an inseparable part of garnering international support and creating a “world people’s front” in solidarity with the righteous cause of the Vietnamese people.

Throughout the two-decade resistance, diplomatic strategies were closely integrated with domestic military and political struggles to foster a favorable international environment. Key diplomatic milestones, such as the 1954 Geneva Conference and the 1973 Paris Peace Accords, demonstrate that diplomacy alongside political and social struggle was an essential factor contributing to the overall success of the Vietnamese revolutionary strategy ( Minh, 2020).

### 1.2 Research problem

Diplomacy during Vietnam’s anti-American resistance war occurred not only at the official level but also encompassed propaganda, international public opinion advocacy, and the mobilization of support from progressive people's movements worldwide. Scholars have previously highlighted the role of people’s diplomacy in connecting the international peace movement with the national liberation cause, thereby consolidating the status and the “just cause” (righteous) perspective of the Vietnamese people on the international stage. Recent studies also indicate that from the era of General Secretary Le Duan onward, the line of people's diplomacy was particularly prioritized in building international relations to create a synergistic strength supporting the Vietnamese revolutionary cause ( Tam and Quang, 2025).

In official discourse, revolutionary diplomacy is understood as a set of formal and informal diplomatic activities aimed at securing political, moral, and material support for the liberation struggle, grounded in the ideology of justice and the progressive social values of the revolutionary movement. The synergy between formal foreign policy strategies and “soft” activities such as external propaganda, engagement with peace movements, and international public opinion created a multi-dimensional diplomatic landscape during the resistance ( Le, 2025).

However, among these studies, few works have thoroughly examined the role of political and ideological work at the grassroots level, particularly in revolutionary battlefields like the Southern region, in constructing and disseminating the Vietnamese revolutionary image to serve these foreign affairs activities. This presents a significant research gap that this article aims to address, clarifying the link between internal political-ideological work and revolutionary foreign affairs operations on the battlefield.

### 1.3 Research gap

While there are numerous works and studies on Vietnam’s revolutionary diplomatic line during the resistance war, the majority focus on the role of formal diplomacy, international agreements, and the negotiation process at the diplomatic table such as the development of diplomacy linked to historic victories from the 1954 Geneva Accords to the 1973 Paris Peace Accords, which created significant achievements on the foreign affairs front ( Son, 2025).

These studies primarily adopt a state-centric perspective, focusing on negotiation tables and bilateral relations. However, they have not yet deeply explored the connection between these diplomatic activities and the “soft” political-ideological activities at the battlefield level the very place where international images and perceptions of the revolutionary movement were nurtured and disseminated through various informal channels ( Le, 2025).

Furthermore, within the study of international relations and diplomacy specifically regarding the concepts of people's diplomacy or public diplomacy although the role of informal activities in mobilizing international public opinion has been recognized, there remains a lack of specific works investigating the role of political-ideological work in battlefields like the Southern theater in constructing and projecting the image of the Vietnamese Revolution to the world ( Vu, 2024).

This represents a critical academic gap, as these battlefields served not only as combat rears but also as cultural, social, and ideological spaces that cultivated the values of a “just cause,” which could influence Vietnam’s legitimacy and soft power on the global stage. Therefore, this study aims to fill that gap by analyzing the role of political-ideological work in the Southern battlefield in building a revolutionary image to serve revolutionary diplomacy, thereby connecting local history with the broader landscape of international relations during the resistance war.

### 1.4 Research questions and central argument

Based on the aforementioned context and research gap, this article aims to analyze the role of political-ideological work in the Southern battlefield in constructing the image of the Vietnamese Revolution for foreign affairs between 1954 and 1975. The specific objective is to clarify how internal activities at the battlefield including propaganda, political education, and socio-cultural activities contributed to the formation of symbols, values, and positive perceptions of the Vietnamese Revolution in international public opinion, thereby impacting the overall effectiveness of revolutionary diplomacy. To achieve this goal, the paper poses several core research questions:

**Question 1:** How was political and ideological work in the Southern battlefield organized and implemented during the 1954–1975 period?

**Question 2:** In what ways did the content and methods of this work contribute to shaping the image of the Vietnamese Revolution across international media channels and public opinion?

**Question 3:** How did the construction of that image impact revolutionary foreign affairs and the mobilization of international support during the resistance?

The central argument of this paper posits that political-ideological work in the Southern battlefield did not merely serve an internal role in building and consolidating revolutionary strength; it also functioned as a vital resource in constructing the righteous and legitimate image of the Vietnamese Revolution internationally, thus indirectly contributing to the efficacy of revolutionary diplomacy. This perspective places battlefield ideological work within a diverse network of methods for mobilizing and securing international support, moving beyond the traditional framework that views such activities solely as internal matters.

This analysis is based on an interdisciplinary approach integrating history, politics, and international relations utilizing concepts such as national image, legitimacy, and soft power to connect battlefield activities with broader foreign policy goals. Within the context where Vietnamese revolutionary diplomacy is viewed as a “strategic front” combining formal diplomacy and soft activities, this research contributes to a deeper understanding of the internal factors that underpinned that diplomatic success ( Son, 2021).

### 1.5 Scientific contributions and article structure

This study offers several notable academic and practical contributions. *First,* the article provides an interdisciplinary perspective that connects revolutionary history and political-ideological work with international relations theories on image, legitimacy, and soft power. In doing so, it expands the scope of research on Vietnamese revolutionary diplomacy beyond the confines of state diplomacy and formal negotiations. *Second,* by focusing on the Southern battlefield as a specific socio-political space, this research elucidates the role of grassroots levels in shaping and disseminating the revolutionary image, thereby emphasizing the multi-layered and multi-actor nature of foreign affairs during a war of national liberation. Practically, the research findings provide a historical and theoretical basis for a deeper understanding of the soft power resources that constituted the diplomatic strength of the Vietnamese Revolution in the 20 ^th^ century.

Regarding the structure, in addition to the introduction and conclusion, the article consists of four main sections: Section 1 presents the historical context and the theoretical framework regarding image, legitimacy, and soft power in international relations; Section 2 analyzes the characteristics and primary content of political and ideological work in the Southern battlefield during the 1954–1975 period; Section 3 clarifies the mechanisms and pathways through which political-ideological work contributed to building the image of the Vietnamese Revolution across foreign affairs channels and international public opinion; Section 4 discusses the theoretical and practical implications of the research findings for the perception of revolutionary diplomacy and soft power within the context of national liberation wars.

## 2. Theoretical foundation and analytical framework

### 2.1 Image, legitimacy, and soft power in international relations

In modern international relations research, “image”, “legitimacy” and “soft power” are considered three central concepts for analyzing the non-material power and influence of actors within the global environment. Diverging from traditional approaches that emphasize military and economic strength, contemporary studies increasingly focus on the roles of perception, discourse, and persuasion in shaping the international status and influence of a state or political movement ( Nye, 2004; Gilpin, 1981).

The concept of “soft power” was introduced by Joseph Nye in the late 1980s to describe the ability to obtain preferred outcomes through attraction and persuasion rather than coercion or payment ( Nye, 2004). According to Nye, the sources of soft power primarily stem from three factors: culture, political values, and foreign policy. Within this framework, a nation's image plays a particularly crucial role in building trust and goodwill within the international community. In the context of conflict and war, soft power not only expands diplomatic space but also strengthens international support for a combatant's objectives and their “just cause”.

Closely linked to soft power is the issue of “image” in international relations. An image does not merely reflect an objective reality; rather, it is the result of a social construction process mediated through discourse, symbols, and political practices ( Jervis, 1976; Wendt, 1999). For national liberation movements, constructing an image as a force representing the aspirations for independence, justice, and social progress is of pivotal importance in garnering the support of international public opinion, peace movements, and progressive forces worldwide. Therefore, a revolutionary image is not just a propaganda tool; it is a constituent element of soft power in the diplomatic struggle.

Parallel to image is the concept of “legitimacy”, understood as the degree to which a political actor is recognized as lawful, justified, and worthy of representing the interests of a specific community ( Beetham, 1991). In international relations, legitimacy pertains not only to legal status but also to moral and political recognition from the international community ( Hurd, 1999). For wars of national liberation, asserting legitimacy as a righteous struggle against oppression and aggression becomes the foundation for expanding foreign relations, mobilizing international support, and undermining the moral standing of the adversary.

From the perspectives of public diplomacy and people’s diplomacy, numerous studies indicate that image and legitimacy do not emerge spontaneously. Instead, they are the results of organized political-ideological strategies deployed through propaganda, political education, cultural activities, and international exchange ( Cull, 2008; Snow & Taylor, 2008). In the context of revolutionary warfare, political and ideological work serves not only internal mobilization but also contributes to the creation of a foreign policy discourse. This, in turn, shapes the international community's perception of the nature, goals, and righteousness of the struggle. At this intersection, image, legitimacy, and soft power become effective analytical tools for studying the external diplomatic role of political-ideological work within revolutionary battlefields.

### 2.2 Political and ideological work as a soft power resource in revolutionary warfare

In classical studies of soft power, soft resources are primarily identified as a nation's culture, political values, and foreign policy ( Nye, 2004). However, in the context of revolutionary warfare and national liberation struggles, soft power is not derived solely from formal state institutions. It is also forged through socio-political practices at the grassroots level specifically, political and ideological work within revolutionary base areas and battlefields. It is through political education, propaganda, the cultivation of cultural life, and mass mobilization that a revolutionary movement constructs a value system, an image, and a foundation of legitimacy capable of resonating beyond national borders.

From a theoretical standpoint, political and ideological work can be approached as a form of “endogenous diplomacy”. In this sense, shaping internal perceptions, beliefs, and political identity serves as the prerequisite for creating external attraction. Social constructivist research in international relations indicates that internal identity and discourse do not just dictate foreign policy behavior; they directly influence how an actor is perceived by the international community ( Wendt, 1999). Consequently, political-ideological work is not merely a tool for internal thought management but a process of constructing strategic soft power resources for foreign affairs.

In revolutionary warfare, political and ideological work performs three key functions directly related to soft power: Disseminating Universal Values: By promoting the ideals of national independence, social justice, and a righteous resistance war, ideological work helps build a value system with universal appeal, aligning with the moral standards and progressive norms recognized by the international community; Founding an Organized Image: By consolidating the faith, discipline, and political mettle of revolutionary forces, ideological work creates a stable foundation for the image of a movement that is organized, idealistic, and capable of legitimately representing national interests; Translating Values into Global Messages: Through cultural activities, journalism, literature, arts, and international exchange, ideological work facilitates the transformation of these values and images into diplomatic messages that resonate with international public opinion.

A distinctive feature of political-ideological work in revolutionary war is the inextricable link between image-building and the assertion of legitimacy. According to Beetham (1991) and Hurd (1999), legitimacy rests not only on legal status but also on moral recognition and social consensus. In the context of armed conflict, demonstrating the “just cause” of the struggle becomes a prerequisite for garnering international support. Political-ideological work, by shaping the discourse on a “just war”, national self-determination, and the anti-aggression nature of the resistance, establishes a solid foundation of legitimacy, thereby elevating the moral standing of the revolutionary movement on the international stage.

From the perspective of public and people's diplomacy, political and ideological work acts as a “soft infrastructure” for foreign affairs. Research in public diplomacy emphasizes that national attraction comes not only from direct diplomatic messaging but is accumulated through domestic social, cultural, and educational practices ( Cull, 2008; Snow and Taylor, 2008). In the case of revolutionary movements, the political-cultural spaces on the battlefield become the birthplaces of symbols, narratives, and role models that exert a powerful persuasive influence on international friends, peace movements, and progressive forces worldwide.

Thus, approaching political-ideological work as a soft power resource allows for an expansion of traditional international relations analytical frameworks. It clarifies the external diplomatic role of grassroots political practices in revolutionary warfare. On this basis, this study views political and ideological work in the Southern battlefield not only as a tool for building internal revolutionary strength but as a vital component in constructing the image, reinforcing the legitimacy, and enhancing the diplomatic efficacy of the Vietnamese Revolution from 1954 to 1975.

## 3. Context and characteristics of the southern battlefield in foreign affairs (1954–1975)

### 3.1 The south in the foreign affairs strategy of the vietnamese revolution (1954–1975)

During the war in Vietnam from 1954 to 1975, diplomacy held a central position in the overall strategy of the Vietnamese Revolution. It aimed to garner international support, expand political space, and assert the legitimacy of the struggle for national reunification. Numerous studies on Vietnamese diplomatic history confirm that diplomacy was not merely a matter of negotiations between governments, but a strategic front that operated in tandem with military and political fronts throughout the entire war ( Bin et al., 2005; Nien et al., 2000). From an international perspective, researchers also emphasize that diplomacy and the mobilization of international public opinion were key components in the overall strategy of the Vietnamese revolutionary movement within the context of the Cold War ( Duiker, 2000; Logevall, 2012).

Within this strategy, the Southern region (Nam Bộ) held a position of particular importance, both geopolitically and diplomatically. Bordering Cambodia and situated at the intersection of mainland Indochina and Southeast Asia, the South became a vital gateway for the Southern revolutionary movement to maintain international communications and connect with regional networks. Vietnamese historical records indicate that the Southern region particularly Saigon-Gia Dinh and the Mekong Delta was a hub for numerous channels of contact with the foreign press, international organizations, and peace movements. This facilitated the transmission of information and images of the resistance to the world ( Editorial Board for the History of the Southern Resistance, 2012). This assessment aligns with international scholarship regarding the role of the South as a unique communicative space between the battlefield and international public opinion ( Herring, 1979; Lawrence, 2007).

Beyond being a key military theater, the South was identified as a critical space for informal diplomacy and international advocacy. Alongside state diplomacy managed by the Central Government and the Ministry of Foreign Affairs, the Southern revolutionary movement deployed various forms of “people-to-people” contact, journalistic exchanges, and the hosting of international delegations, while connecting with peace and humanitarian organizations. Documents from the Vietnamese Ministry of Foreign Affairs show that these channels contributed directly to disseminating political positions, the goal of national reunification, and the “just cause” nature of the struggle ( Bin et al., 2005; Nien et al., 2000). From an international viewpoint, Logevall (2012) affirms that these informal contact channels in the South played a decisive role in shaping global public perception of the war in Vietnam.

Strategically, the South also served as the link between the foreign policy planning centers of the Vietnamese Revolution and the battlefield realities of South Vietnam. Political messages, discourses on “just war”, and moral-legal arguments were implemented and tested through international contacts in this region. Simultaneously, feedback from the foreign press, anti-war movements, and international organizations was channeled back, contributing to the refinement and completion of the international advocacy strategy of the Vietnamese Revolution ( Logevall, 2012).

From the perspective of soft power and image in international relations, it is evident that the role of the South in revolutionary diplomatic strategy was determined not only by military or geographical factors but also by its ability to generate political attraction and moral persuasion for the international community. It was within this space that socio-political practices at the battlefield level became an integral part of foreign policy resources, contributing to the formation of the image and the consolidation of the legitimacy of the Vietnamese revolutionary movement in international perception. This serves as the foundation for a deeper analysis of the role of political and ideological work in the Southern battlefield in the subsequent sections.

### 3.2 Characteristics of the socio-political space in the southern battlefield

The Southern theater (Nam Bộ) during the 1954–1975 period was not merely a military zone but, primarily, a unique socio-political space where the revolutionary movement formed and operated in close synergy with the local populace. Vietnamese historical accounts confirm that the South was a land with a long tradition of struggle, characterized by a diverse social structure including peasants, small merchants, urban intellectuals, religious communities, and ethnic groups. This created a dynamic and highly interactive social environment ( Editorial Board for the History of the Southern Resistance, 2012). This diversity facilitated the formation of broad and flexible mass networks, which served as the vital foundation for political and ideological activities within the battlefield.

A salient feature of the Southern socio-political space was the intricate overlapping of revolutionary base areas with cities, towns, peri-urban zones, and trade routes. This space was not isolated; it maintained constant channels of contact with the outside world, including urban residents, intellectuals, the press, and various religious and social groups. Research on South Vietnamese wartime society indicates that the connectivity between the battlefield and urban centers created a unique communication environment, where information, political discourse, and images of the revolutionary movement were disseminated through multi-directional channels ( Lawrence, 2007). Consequently, the Southern battlefield was not only a site for organizing resistance but also a space for producing and propagating political messages.

Within the social life of the battlefield, mass organization held a central role. Political activities, ideological education, cultural and artistic events, and emulation movements were implemented extensively to consolidate faith, maintain discipline, and generate social consensus. Documents from the War Review Steering Committee emphasize that cultural, educational, and propaganda activities in the South served a dual purpose: they mobilized internal forces and simultaneously projected an image of a stable, organized, and idealistic society, thereby enhancing the political attraction of the revolutionary movement ( Editorial Board for the History of the Southern Resistance, 2012). From an international perspective, Herring (1979) argues that these social practices established a moral foundation that helped the Vietnamese revolutionary movement gain the sympathy of global public opinion.

Furthermore, the Southern battlefield was a space of frequent contact with the foreign press, humanitarian organizations, peace delegations, and international intellectual networks. The presence of these contact channels imbued the socio-political life of the battlefield with a distinct external diplomatic dimension. Logevall (2012) points out that many international reporters and activists directly accessed Southern base areas, subsequently documenting and transmitting images of social life, the spirit of resistance, and the movement’s legitimacy to the international public. The social space of the battlefield thus became a critical “signal transmitter” within the global network of communication and advocacy.

From the analytical lens of soft power, it is evident that the open, diverse, and interactive characteristics of the Southern socio-political space facilitated the creation of symbolic and moral resources. This environment allowed the revolutionary movement to maintain internal cohesion while projecting the image of an organized society, deeply rooted in the people and driven by high ideals. This specific socio-political milieu provided the bedrock for the role of political and ideological work in constructing the Vietnamese revolutionary image to serve foreign affairs.

## 4. The role of political-ideological work in constructing the vietnamese revolutionary image for foreign affairs

### 4.1 Constructing political discourse and the legitimacy of the revolutionary movement

In modern conflicts, the ability to construct and maintain legitimacy plays a pivotal role in determining the diplomatic efficacy and international standing of combatant actors. International Relations theory suggests that alongside military and institutional power, political discourse serves as a central resource for creating legitimacy and shaping international perceptions regarding the nature of a conflict ( Finnemore & Sikkink, 1998; Hurd, 1999). For the Vietnamese revolutionary movement, political and ideological work in the Southern battlefield provided the essential space to form, standardize, and disseminate political discourses that served diplomatic objectives.

A core function of political-ideological work was the construction of a system of moral and legal arguments to assert the movement's legitimacy. Vietnamese theoretical documents emphasize that propaganda and political education on the battlefield were not merely intended for mass mobilization; they were aimed at clarifying the “just cause” of the struggle by linking the goals of independence and unification with universal principles such as national self-determination and state sovereignty ( Nien et al., 2000; Bin et al., 2005). From an IR perspective, aligning revolutionary goals with international norms is viewed as a typical strategy to increase legitimacy and mitigate diplomatic isolation ( Finnemore, 1996; Reus-Smit, 2004).

Within the Southern battlefield, political discourse was not only crafted at the central level but was also operationalized through the network of political officers, ideological education systems, and forms of mass activity. This process produced a coherent messaging system regarding the nature of the war as a struggle for national liberation, a resistance against external imposition, and a movement toward national reunification. Vietnamese historical studies show that the consistency of this discourse reinforced internal conviction and provided a foundation for articulating a coherent message to the outside world. Theoretically, this constitutes a process of “discursive standardization” aimed at establishing a political identity capable of persuading the international community ( Wendt, 1999).

Crucially, the political discourse emerging from the battlefield became a central component of the Vietnamese revolutionary movement's strategy to mobilize international public opinion. Through press contacts, people's diplomacy, and international intellectual networks, messages regarding the “just cause,” the defensive nature of the struggle, and the objective of peace were widely transmitted. This contributed to shaping global public perception of the war in Vietnam ( Logevall, 2012). In the soft power approach, the ability to persuade through moral arguments and normative standards is considered a key resource that enhances political attraction and reduces the costs of coercion ( Nye, 2004).

From the perspective of legitimacy analysis, it is evident that political-ideological work in the Southern battlefield contributed significantly to projecting an image of a revolutionary movement that represented national interests while remaining consistent with the universal values of the international community. The synergy between the discourse of national liberation and the language of international norms allowed the Vietnamese revolutionary movement to gradually transition from a mere combatant actor into a prestigious and widely recognized political entity ( Hurd, 1999). In this sense, political and ideological work was not just an internal mobilization tool but a foundational component of the foreign affairs strategy and soft power in the Vietnamese revolutionary war.

### 4.2 Culture, education, and social life as tools for revolutionary image-building

In International Relations (IR) scholarship, culture and social life are regarded as vital channels of soft power, through which political actors generate attraction and shape their image within international perception ( Nye, 2004; Cull, 2008). Far from being limited to peacetime, research indicates that in the context of war, socio-cultural practices can become effective instruments for constructing a political image, reinforcing legitimacy, and fostering international sympathy ( Lawrence, 2007). For the Vietnamese revolutionary movement, political and ideological work in the Southern battlefield deployed extensive cultural, educational, and social organizational activities as an organic component of its image-building strategy for foreign affairs.

A prominent feature of the Southern battlefield was the seamless integration of political education with socio-cultural life. Activities such as political study, propaganda, literature, arts, and journalism were intended not only to maintain the spirit of resistance but also to project the image of an organized, disciplined society deeply rooted in the people. Vietnamese historical records emphasize that cultural life on the battlefield was organized to promote revolutionary ethics, community spirit, and a modest lifestyle, thereby creating a system of symbols representing the ideal of national liberation ( Editorial Board for the History of the Southern Resistance, 2012). From a soft power perspective, this represents the creation of “moral attraction”, where the image of an idealistic and disciplined society becomes a persuasive resource for international public opinion ( Nye, 2004).

Resistance culture in the South also functioned as a medium for transmitting the revolutionary image through art, journalism, and exchange. Revolutionary literary, musical, and theatrical works met internal spiritual needs while simultaneously building a symbolic system of Vietnamese people and society during the war. International studies on the Vietnam War show that these cultural symbols and images of social life helped the revolutionary movement establish a distinct identity one that contrasted with purely military discourses by emphasizing the humanistic and moral dimensions of the struggle ( Lawrence, 2007). In IR terms, this constitutes a form of “symbolic power,” allowing a combatant to increase attraction without resorting to coercion ( Bourdieu, 1991).

Furthermore, organizing social life on the battlefield to be stable, disciplined, and people-centric facilitated international engagement. Foreign correspondents, peace activists, and humanitarian organizations accessing the battlefield documented not only military activities but also social life, cultural practices, and community spirit. These observations, reflected in international press and memoirs, helped disseminate the image of a revolutionary movement with a broad social base and organizational capacity, thereby strengthening the persuasiveness of Vietnam's political message on the global stage ( Logevall, 2012).

From an image and soft power analysis perspective, the socio-cultural and educational practices in the Southern battlefield created a unique “symbolic space”. Here, the revolutionary image was constructed through moral values, community spirit, and the ideal of national liberation. This space not only bolstered internal cohesion but also provided a critical visual and narrative resource for foreign affairs, shaping international recognition of the Vietnamese revolutionary movement as a political actor with profound social depth and a solid moral foundation.

### 4.3 Mass mobilization and people’s diplomacy from the southern battlefield

In contemporary IR theory, people’s diplomacy and public diplomacy are viewed as essential channels supplementing state diplomacy, particularly in contexts of conflict and ideological competition ( Cull, 2008; Melissen, 2011). Research on the Vietnam War indicates that alongside formal negotiations, direct engagement with international society and public opinion played an increasingly vital role in shaping perceptions of the war's nature and legitimacy ( Logevall, 2012). Within this framework, the Southern battlefield emerged as a specific site for people’s diplomacy, where political-ideological work was inextricably linked to international contact and public opinion advocacy.

A notable characteristic of the Southern battlefield was the relatively frequent presence of foreign journalists, humanitarian organizations, and international peace delegations. Through the network of mass organizations and political cadres, the revolutionary movement organized various forms of reception, guidance, and information sharing, enabling these actors to directly experience social life and wartime realities. Vietnamese historical documents note that the selection of contact spaces, the organization of daily activities, and the content of discussions were meticulously prepared to ensure the consistency of the political message and the social image transmitted abroad ( Editorial Board for the History of the Southern Resistance, 2012; Bin et al., 2005). In public diplomacy terms, this is a form of “engagement management” designed to orient international perception through direct experience ( Cull, 2008).

In the practical context of the South, mass mobilization served as a key intermediary between the battlefield and informal international partners. Propaganda, mobilization cadres, and local grassroots networks managed internal life while also participating directly in communicating political stances, explaining struggle objectives, and responding to inquiries from international scholars and activists. Many international memoirs and studies reveal that these grassroots encounters within daily environments created a strong impression of discipline, community spirit, and social support for the revolution ( Lawrence, 2007). In IR approaches, this is categorized as “face-to-face diplomacy”, where social experience carries more weight than official declarations ( Melissen, 2011).

Another significant dimension was the Southern movement's proactive connection with transnational peace movements, humanitarian organizations, and progressive intellectuals. Through these channels, information regarding social life, the war situation, and political stances reached international public opinion outside official diplomatic channels. Logevall (2012) points out that these transnational social networks were instrumental in forming the anti-war movement and exerting political pressure on governments involved in the conflict. In the case of the South, grassroots political-ideological work was pivotal in maintaining message consistency and ensuring the credibility of these contact channels.

From a soft power analysis, people’s diplomacy within the Southern battlefield did not only expand the scope of international contact but also created a unique mechanism to transform social resources into foreign policy assets. Through direct contact practices and political messaging, the revolutionary movement progressively built an image of a force with a broad social foundation, high organizational capacity, and profound legitimacy. This served as a vital prerequisite for the sustainable dissemination of the Vietnamese revolutionary image, enhancing diplomatic efficacy and supporting the subsequent processes of international negotiation and advocacy.

### 4.4 External impact: From the battlefield space to international public opinion and perception

In international relations, the external impact of an actor is measured not only by negotiation outcomes or specific policy changes but also profoundly by the ability to shape the perceptions, norms, and interpretive frameworks of the international community regarding a conflict and its combatants ( Finnemore & Sikkink, 1998; Hurd, 1999). In the case of the Vietnamese Revolution, the political-ideological practices deployed from the Southern battlefield created a chain of indirect yet sustainable impacts on global perception, establishing a strategic foundation of image and legitimacy for foreign affairs.

**First,** by constructing a consistent political discourse, building a morally grounded social image, and broadly implementing people’s diplomacy, the Vietnamese revolutionary movement gradually shaped an international cognitive framework. In this framework, the war was perceived as a legitimate struggle for national liberation rather than a purely ideological conflict. International research on the Vietnam War indicates that shifts in the discourse of the Western press, academia, and anti-war movements from the late 1960s increasingly reflected this approach ( Herring, 1979; Logevall, 2012). Within the soft power analytical framework, this is a process of “cognitive restructuring”, a central mechanism that helps a combatant expand political space and minimize the costs of confrontation ( Nye, 2004).

**Second,** the external impact of political-ideological work was evidenced in the consolidation and expansion of the international legitimacy of the Vietnamese revolutionary movement. By synthesizing national liberation discourse with international normative language regarding self-determination, peace, and justice, the movement transitioned from an armed force into a widely recognized political actor. Research on legitimacy in international relations indicates that such recognition depends not only on military capability but also on alignment with the prevailing norms of the contemporary international order ( Hurd, 1999; Reus-Smit, 2004). In Vietnam’s case, battlefield political-ideological practices helped generate “legitimacy capital”, a key resource supporting subsequent negotiations and international advocacy.

**Third,** from a soft power perspective, the Southern battlefield operated as a unique “soft power infrastructure” within a wartime context. Through mass networks, cultural-educational activities, and people’s diplomacy channels, the movement transformed social and moral resources into cross-border political attraction. Unlike peacetime soft power, which relies heavily on institutions, economics, and popular culture, soft power in this case was constructed primarily from moral image, national representation, and social organizational capacity under wartime conditions. This is a form of “contextualized soft power”, where war does not extinguish but rather highlights the symbolic value and moral persuasiveness of the combatant ( Bourdieu, 1991; Nye, 2004).

Theoretically, the case of the Southern battlefield demonstrates that political-ideological work is not just an internal governance tool but a foreign affairs mechanism capable of creating long-term cognitive impact. By connecting discourse, social image, and people’s diplomacy, the Vietnamese Revolution formed a model for operating soft power during war, where grassroots social space becomes an active agent of foreign policy. This approach supplements existing theories of soft power and public diplomacy by highlighting the role of non-state socio-political practices in constructing the international image and legitimacy of revolutionary movements.

## 5. Conclusion

This article has analyzed the role of political and ideological work in the Southern battlefield in constructing the image of the Vietnamese Revolution for foreign affairs during the 1954–1975 period, thereby illuminating a neglected dimension in war and international relations studies. By connecting grassroots social space with the construction of image, legitimacy, and soft power, the research demonstrates that political-ideological work is not only an internal management tool but also a foreign policy resource with a lasting cognitive impact on the international community.

Theoretically, the case of the Southern battlefield supplements current approaches to soft power and public diplomacy by highlighting the role of socio-political practices in revolutionary warfare. Unlike peacetime models, soft power here was derived from moral image, national representation, and grassroots organizational capacity. This suggests that soft power is not solely a product of the modern state but can be forged by revolutionary movements during prolonged conflict.

In terms of historical practice, the research confirms that the Southern battlefield operated as a specific socio-political infrastructure, wherein political-ideological work generated “image capital” and “legitimacy capital” for the Vietnamese Revolution on the world stage. These practices directly supported people’s diplomacy and indirectly expanded the space for international negotiation, enhancing the overall efficacy of the wartime foreign policy strategy.

Broadly, the article suggests an integrated approach between war history and international relations, where grassroots social space is viewed as a significant foreign policy actor. This approach can be applied to study other 20 ^th^ century national liberation movements and non-traditional forms of soft power in contexts of conflict and political transition. Future research could expand this analysis through comparative studies of different battlefields or revolutionary movements in Southeast Asia and beyond, further exploring the relationship between ideological work, people’s diplomacy, and the formation of international public opinion in modern warfare.

### Ethical considerations

No ethical approval or consent is required for this study.

## Data Availability

No new data were created or analyzed in this study. This article relies solely on publicly available sources cited in the reference list.
