# Supplementary material for: Political and Ideological Work in the Southern Battlefield and the Construction of the Vietnamese Revolutionary Image in Foreign Affairs (1954–1975)
Source: F1000Res. 2026 Mar 13;15:399. [Version 1] doi: 10.12688/f1000research.178080.1 (PMC13040224; doi:10.12688/f1000research.178080.1)
Supplement: Peer review history

# Reviewer report: Reviewer response for version 1

**DOI:** 10.5256/f1000research.196420.r468146

**Published:** 2026-03-31

**Authors:** Dang Thi Minh Phuong

**Affiliations:** 1 Industrial Unversity of Ho Chi Minh City, Ho Chi Minh City, Vietnam

*General Assessment:*

 The manuscript presents high-quality content with a modern methodological approach, offering significant original contributions to the fields of diplomatic history and international relations. The author demonstrates a seamless integration of traditional historical documentation with contemporary theoretical frameworks, including “soft power,” “legitimacy,” and “national image.”

*Key Strengths:*

 Originality and Significance: The study effectively addresses a notable research gap by establishing a critical link between grassroots political and ideological work (specifically within the Southern battlefield) and the efficacy of revolutionary diplomacy.

 Robust Theoretical Framework: The application of Joseph Nye’s soft power concepts alongside constructivist perspectives in international relations provides the paper with profound academic depth and high international integration potential.

 Logical Argumentation: The author offers a persuasive analysis of how localized cultural, educational, and mass mobilization activities were transformed into “image capital” and “legitimacy capital,” thereby exerting a strong attraction on global public opinion and international peace movements.

 Comprehensive Source Material: The manuscript utilizes a diverse array of sources, ranging from official Party documents and memoirs to prestigious international scholarly works, ensuring a multifaceted and well-rounded perspective. 

*Recommendations:*

 Consider further elucidating the bidirectional interaction (feedback loop) between international public opinion and the subsequent policy adjustments on the battlefield.

 The inclusion of a communication flow diagram could potentially enhance the visual clarity and structural presentation of the research.

*Conclusion:*

 The manuscript fully satisfies the criteria for high-quality academic research. The arguments are articulated clearly, the structure is coherent, and the findings hold substantial practical significance.

Is the work clearly and accurately presented and does it cite the current literature?

Yes

If applicable, is the statistical analysis and its interpretation appropriate?

Yes

Are all the source data underlying the results available to ensure full reproducibility?

Yes

Is the study design appropriate and is the work technically sound?

Yes

Are the conclusions drawn adequately supported by the results?

Yes

Are sufficient details of methods and analysis provided to allow replication by others?

Yes

Reviewer Expertise:

Vietnamese History, Political Science, Public Administration Reform, Digital Transformation.

I confirm that I have read this submission and believe that I have an appropriate level of expertise to confirm that it is of an acceptable scientific standard.

# Reviewer report: Reviewer response for version 1

**DOI:** 10.5256/f1000research.196420.r468148

**Published:** 2026-03-23

**Authors:** Quoc Dung Mai

**Affiliations:** 1 Ho Chi Minh City University of Industry and Trade, Ho Chi Minh City, Ho Chi Minh City, Vietnam

Strengths:

 The concept of “endogenous diplomacy” as a prerequisite for external attraction is a particularly profound theoretical contribution.

 The analysis in Section 4.2 regarding how the “resistance culture” and daily social life on the battlefield acted as a “moral attraction” for foreign observers is highly persuasive.

 The paper clearly illustrates how the Southern battlefield functioned as a “signal transmitter” to the global peace movement through direct contact and people's diplomacy.

 Minor Suggestion (Optional):

 The author might consider briefly mentioning any specific challenges or “counter-propaganda” efforts from the adversary that the ideological work had to overcome. This would add an extra layer of complexity to the analysis of the battle for international public opinion.

Is the work clearly and accurately presented and does it cite the current literature?

Yes

If applicable, is the statistical analysis and its interpretation appropriate?

Partly

Are all the source data underlying the results available to ensure full reproducibility?

Yes

Is the study design appropriate and is the work technically sound?

Yes

Are the conclusions drawn adequately supported by the results?

Yes

Are sufficient details of methods and analysis provided to allow replication by others?

Partly

Reviewer Expertise:

International Relations, Area Studies, Historiography, Political Science

I confirm that I have read this submission and believe that I have an appropriate level of expertise to confirm that it is of an acceptable scientific standard.
